# Transient Receptor Potential (TRP) Channels as Fundamental Regulators of Fibrosis and Pruritus—A New Therapeutic Target for Pathological Scar Management

**DOI:** 10.3390/ijms27020815

**Published:** 2026-01-14

**Authors:** Yuchen Tang, Zheng Zhang, Yixin Zhang

**Affiliations:** Department of Plastic and Reconstructive Surgery, Shanghai Ninth People’s Hospital, School of Medicine, Shanghai Jiao Tong University, Shanghai 200011, China; yuchentang_aysha@163.com

**Keywords:** transient receptor potential channels, pathological scars, fibrosis, pruritus, targeted therapy, pharmacotherapy

## Abstract

Pathological scars (PSs), which encompass hypertrophic scars (HSs and keloids, pose significant challenges in the realm of plastic surgery due to their characteristics of excessive fibrosis and persistent pruritus. This fibrosis can lead to both functional limitations and aesthetic issues, while pruritus often indicates ongoing scar development and greatly impacts quality of life. Although the underlying cause of both conditions is linked to dysregulated inflammation, the specific connections between fibrosis and pruritus are not well understood. Transient receptor potential channels (TRP), known for their roles in systemic fibrotic diseases and as mediators of chronic pruritus in skin disorders, may play a crucial role in the environment of pathological scars. This review compiles existing research to investigate the idea that certain TRP subfamilies (TRPA1, TRPV1, TRPV3, TRPV4) could link fibrosis and pruritus in pathological scars by interacting with common inflammatory mediators. We suggest that these channels might act as central molecular hubs that connect the signaling pathways of fibrosis and pruritus in these scars. Therefore, targeting TRP channels pharmacologically could be a promising approach to simultaneously alleviate both fibrosis and pruritus, potentially leading to a new dual-pathway treatment strategy for managing pathological scars. Our review also critically examines the current landscape of TRP-targeted therapies, pointing out challenges such as limited selectivity for specific subtypes and the lack of clinical trials focused on pathological scars, while emphasizing the necessity for interdisciplinary advancements in this area. In conclusion, while TRP channels are attractive targets for therapeutic intervention in pathological scars, their effective clinical application necessitates a more profound understanding of the mechanisms specific to scars and the creation of targeted delivery methods.

## 1. Introduction

Pathological scars (PSs), which encompass hypertrophic scars (HSs) and keloids, result from an overproduction of extracellular matrix (ECM) due to imbalanced fibrotic signaling during the healing of skin wounds [1]. While not life-threatening, PS can lead to both aesthetic and functional challenges, with symptoms like pruritus and pain significantly impacting patients’ overall well-being and mental health. Research indicates that between 67% and 95% of individuals with keloids report experiencing pruritus [2,3,4], while 57% to 100% of burn victims with HS also report similar symptoms [5]. Pruritus can occur at various stages of scar formation, but clinical evidence suggests that it becomes particularly intense and frequent during the proliferative phase of PS, making it an important clinical marker for doctors assessing scar hyperplasia. Consequently, established scar assessment tools, such as POSAS (Patient and Observer Scar Assessment Scale) [6], incorporate pruritus scores as a measure of scar severity, highlighting the link between pruritus and the advancement of scar fibrosis. Despite the strong correlation between PS and pruritus, there is a lack of extensive foundational studies that explain or propose theories regarding this relationship.

PS pathology is characterized by an overgrowth of various cellular elements, such as fibroblasts (FBs), vascular endothelial cells, and inflammatory cells. The infiltration of these inflammatory cells is influenced by a range of cytokines and chemokines, which play a role in the development of PS [7]. Additionally, the pruritus associated with scars involves not just sensory neurons but also a variety of other cells, including keratinocytes (KCs), FBs, mast cells, macrophages, and other immune cells such as T lymphocytes and eosinophils, creating a complex biological network. In clinical settings, the primary treatment for PS is intralesional corticosteroids, known for their anti-fibrotic and anti-inflammatory properties, which may help relieve scar-related pruritus. This indicates a possible interconnected system that links fibrosis and pruritus. Understanding this triadic relationship could offer new insights into the progression of PS and aid in the creation of targeted treatment strategies [8,9].

Recently, transient receptor potential (TRP) channels have gained significant attention in research related to both pruritus [10,11] and fibrosis [9,12,13], revealing shared molecular mechanisms that link dermal fibrosis with pruritus. This study aims to explore the involvement of TRP channels in these conditions and their potential as biomarkers or therapeutic targets for pruritus and fibrosis associated with PS.

## 2. Classification and Function of TRP Channels in Skin

TRP channels are non-selective cation channels and are found in the membranes of plasma or intracellular organelles. In mammals, the TRP superfamily is divided into six subfamilies based on sequence homology: TRPC (canonical), TRPV (vanilloid), TRPM (melastatin), TRPA (ankyrin), TRPML (mucolipin), and TRPP (polycystin) [11].

TRP channels are extensively found in various skin cells, including sensory neurons, KCs, FBs, melanocytes, and immune cells such as macrophages and mast cells. Functionally, they regulate physiological processes such as thermosensation [14], barrier homeostasis [15], and hair growth [16], while also contributing to pathological conditions like inflammatory responses, fibrotic remodeling, and chronic pruritus. Numerous research efforts have investigated the impact of TRP channels on skin fibrosis and pruritus [10,12,13]. Additionally, TRP channels possess pharmacologically accessible sites and offer the potential for drug development, underscoring their important potential in clinical applications [17].

## 3. PS Fibrosis and TRP Channels

### 3.1. FBs as the Main Effector Cells of Dermal Fibrosis

PS is marked by an abnormal and ongoing accumulation of ECM, primarily resulting from the overactivity of dermal FBs, which are the key effector cells in fibrogenesis [18,19]. Normally, FBs are involved in tissue repair through their proliferation, migration, and ECM synthesis. However, in PSs, this regulatory process is disrupted. Certain FBs differentiate into myofibroblasts (MFBs), which are distinguished by the expression of alpha-smooth muscle actin (α-SMA) and show increased contractile function and ECM production [20]. The continuous activation of both FBs and MFBs in PSs is maintained by a complex and enduring pathological environment that encompasses various activating factors, such as fibrogenic growth factors, immune responses, and mechanical stress [7,21].

The development of fibrosis is largely influenced by various fibrogenic growth factors, including transforming growth factor-β (TGF-β), fibroblast growth factor, platelet-derived growth factor, vascular endothelial growth factor, and periostin. TGF-β, predominantly released by FBs, is recognized as the key regulator of dermal fibrosis and has interactions with TRP channels [22]. The classic form of TGF-β1 can stimulate the expression of TRPA1 and TRPC6 through the p38/mitogen-activated protein kinase (MAPK)/serum response factor (SRF) signaling pathway, which activates downstream pathways involving calcium/calmodulin-dependent protein kinase and nuclear factor of activated T cells (NFAT), leading to calcium influx and FB differentiation, thereby increasing the levels of α-SMA [13,23]. Furthermore, the upregulation of TRPM7 triggers the phosphatidylinositol 3-kinase (PI3K)/protein kinase B (AKT) pathway, which boosts the expression of TGF-β1 and α-SMA, facilitating collagen accumulation in vitro in HS [24].

In addition to growth factors, immune system imbalances represent another key aspect of the pathological microenvironment. Infiltrating immune cells (e.g., macrophages, T lymphocytes) release these mediators, perpetuating a cycle of activation. A shift towards pro-fibrotic M2 macrophages and a Th2-dominant response are often observed. Evidence suggests that neurogenic factors like SP and CGRP can influence FB activity. Sustained tension is a critical driver of FB activation and ECM remodeling in PS [25,26,27].

Ultimately, the mechanical tension within the microenvironment plays a vital role in activating FBs and facilitating ECM remodeling in PS [28]. Research indicates that neurogenic elements such as substance P (SP) and calcitonin gene-related peptide (CGRP) can influence FB behavior [29]. Notably, TRP channels are emerging as pivotal signaling hubs that sense and transduce these diverse microenvironmental signals. They act as integrative platforms for inflammatory, chemical, and mechanical stimuli, ultimately modulating FB function and determining the fibrotic outcome, a theme that will be elaborated upon in the following section.

### 3.2. TRP Channels Mediate KC-FB Interactions in Dermal Fibrosis

Recent studies underscore the significance of interactions between KCs and FBs as key contributors to dermal fibrosis, with certain TRP channels serving as molecular intermediaries [30]. The stimulation of TRPA1 in primary human KCs leads to the release of interleukin-1 (IL-1), which can initiate the production of fibrogenic factors (such as keratinocyte growth factor, granulocyte-macrophage colony-stimulating factor, and transforming growth factor-alpha) and inflammatory substances (including IL-6, IL-8, cyclooxygenase-2, and prostaglandin E2) through paracrine mechanisms [31]. Inhibitors of TRPV2, like SKF96365 and tranilast, reduce TGF-β1 secretion from KCs, thereby diminishing FB differentiation in both in vivo and in vitro models of wound healing in rats [22]. Thymic stromal lymphopoietin (TSLP), primarily secreted by KCs and FBs, plays a vital role in Th2 immune responses. The activation of TRPV3 by compounds such as carvacrol enhances TSLP/Smad2/3 signaling in FBs derived from HSs, facilitating collagen accumulation [32]. At the same time, TRPV3 activation in KCs intensifies inflammation mediated by NF-κB, which may further amplify TGF-β1 signaling [33]. An overactive epithelial-mesenchymal transition (EMT) process persistently converts epithelial cells into FBs or MFBs, leading to excessive ECM accumulation [34]. There is evidence that KCs in psoriatic skin may experience a partial EMT-like transformation. In keloids, TGF-β1 is known to influence this EMT-like characteristic, although the direct conversion of KCs into FBs or MFBs has yet to be established [35]. In a model of skin fibrosis induced by bleomycin, TRPV4 is implicated in mediating TGF-β1-induced EMT through the activation of Yes-Associated Protein/Transcriptional Co-activator with PDZ-Binding Motif (YAP/TAZ) and PI3K/AKT pathways [36]. While the involvement of TRPV4 in EMT within human PS necessitates further direct investigation, it presents a promising area for future research.

### 3.3. TRP Channels Mediate Immunocytes and Neuroimmune Interactions

Recent findings indicate that TRP channels may play a role in the dysregulation of immune cells associated with dermal fibrosis. In studies involving mice lacking TRPA1, there was a notable decrease in the infiltration of FBs and T cells, along with a reduction in the levels of α-SMA and COL-1 during the healing process [37]. In models of scleroderma and atopic dermatitis, cytokines IL-4 and IL-13, produced by Th2 cells, were found to enhance the expression of TRPA1 and TRPV1 [12,38]. These cytokines further promote fibrosis by increasing periostin levels, which in turn triggers RhoA/ROCK-mediated secretion of TGF-β1 from dermal FBs, creating a self-perpetuating cycle [39]. Additionally, TSLP, which stimulates Th2 immune responses and boosts COL-1/COL-3 synthesis through TGF-β1/CXCR4/SDF-1α signaling in FBs derived from human keloids, showed a positive correlation with TRPA1 levels [40]. The activation of TRPV1 leads to the release of neuropeptides such as SP and CGRP. CGRP intensifies fibrosis by fostering Th17 immune responses and the secretion of IL-17 and IL-22, while antagonists of CGRP can diminish the expression of COL-1, TGF-β1, and α-SMA in fibroblasts from HSs and mitigate inflammation from macrophages through NF-κB/ERK signaling pathways [29]. This points to a possible neuroimmune-fibrotic connection in which TRPV1 has a significant role.

### 3.4. TRP Channels Mediate Mechanical Force-Induced Dermal Fibrosis

Mechanical force is a fundamental driver of PS pathogenesis, with various TRP channels acting as essential mechanosensors [28].Sustained mechanical tension directly leads to an increase in TRPC3 levels in FBs, triggering the Smad3/NF-κB signaling pathway, which boosts the production of COL-1, TGF-β1, α-SMA, and fibronectin in HS models [41]. Additionally, mechanical stress also induces KC-derived endothelin-1 (ET-1), which binds to endothelin receptor B (EDNRB) on FBs, further enhancing TRPC3 expression and fibrotic activity [42]. TRPC6 overexpression promotes FB differentiation via the calcineurin/NFAT pathway, exacerbating collagen deposition in vitro and in vivo wound healing models [43]. Moreover, the mechanosensitive channel TRPV4 forms a complex with Piezo1 upon activation, generating a sustained Ca^2+^ signal that enhances mechanical signaling and intensifies fibrotic processes in HSs [44].

**Figure 1:**
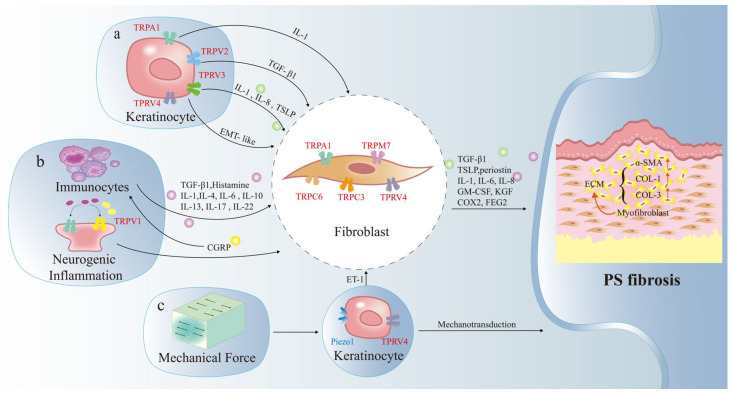
Mechanism network of TRP channels in pathogenic dermal fibrosis. FB differentiate into MFBs, accompanied by excessive secretion of COL-1, COL-3, and a-SMA, eventually leading to PS fibrosis. KCs (a), immunocytes (b), and mechanical force (c) are involved in the upstream regulation of dermal fibrosis, with various TRP channels (such as TRPA1, TRPV1, TRPV2, TRPV3, TRPV4, TRPC3, TRPC6, TRPM7) playing different roles. Red arrows indicate increased protein expression. TSLP Thymic stromal lymphopoietin, IL-4 interleukin-4, ET-1 Endothelin-1, EMT epithelial-mesenchymal transition, GM-CSF granulocyte-macrophage colony-stimulating factor, KGF keratinocyte growth factor, COX2 cyclooxygenase-2, TGF-β1 transforming growth factor-β1, PS pathological scars, TRPA1 transient receptor potential A1.

To conclude, TRP channels such as TRPC3, TRPC6, and TRPV4 play a crucial role in mechanotransduction within PS, collaborating effectively with additional sensors to drive the process of fibrotic remodeling (Figure 1).

## 4. PS Pruritus and TRP Channels

### 4.1. Pruritus Originates from Peripheral Pruriceptive Fibers

**Figure 2:**
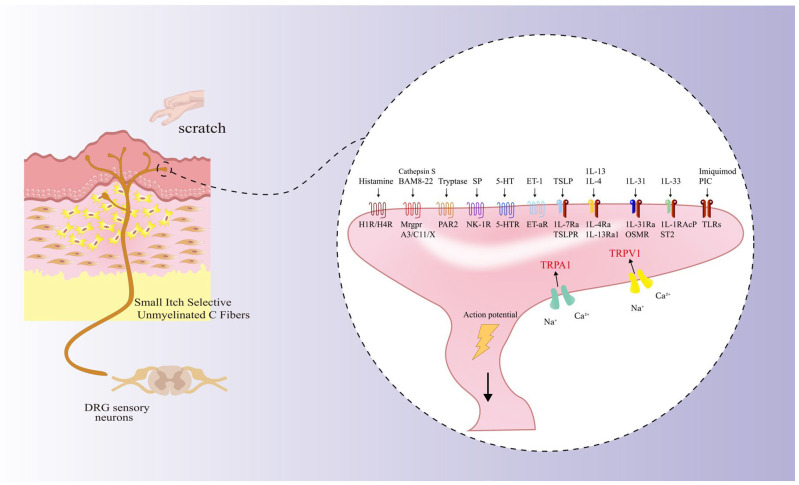
Schematic Diagram of the Peripheral Pruriceptive Signaling Pathway. Pruritogens (e.g., histamine, TSLP, IL-31) bind to their specific receptors located on the peripheral nerve terminals of small unmyelinated C fibers that are selective for itch. These receptors include G protein-coupled receptors, cytokine receptors, and Toll-like receptors. Their activation converges on and opens key cation channels, particularly TRPA1 and TRPV1. This process leads to receptor-operated calcium entry (ROCE), membrane depolarization, and the generation of action potentials. The pruritic signal is subsequently transmitted along the afferent nerve fiber, through the dorsal root ganglion (DRG), to the central nervous system for processing, ultimately eliciting the sensation of pruritus.

Pruritus is a clinical condition marked by a strong and often distressing desire to scratch, which is particularly common among individuals with PSs [19,45]. When pruritus becomes severe or chronic (>6 weeks), it significantly affects a person’s quality of life. The mechanisms behind pruritus involve complex interactions among pruritogens, specialized pruriceptive receptors, afferent nerve fibers, dorsal root ganglia (DRG), and specific areas of the central nervous system. Pruritogens are bioactive compounds released by cells that interact with pruriceptive receptors found on afferent nerve fibers, primarily small-diameter unmyelinated C fibers. These receptors, which include GPCRs, cytokine receptors, and Toll-like receptors (TLRs), facilitate the opening of ion channels, particularly TRP channels like TRPV1 and TRPA1, and mediate receptor-operated calcium entry (ROCE), leading to the generation of pruritus action potentials [46] (Table 1 and Figure 2).

In PS, a multifaceted pruritogenic environment is established, where KCs, FBs, immune cells, and sensory nerves form an interactive network, driving inflammatory pruritus pathophysiology. This type of pruritus arises from the release of various substances during inflammation, primarily from immune-inflammatory cells and peripheral sensory neurons, contributing to neurogenic inflammation in PS. Numerous mediators, including histamine, IL-4, IL-13, IL-31, ET-1, and neuropeptides, contribute to inflammatory pruritus, regardless of their source, whether from immune-inflammatory cells or sensory neurons [60,67]. Importantly, as discussed below, studies indicate that certain TRP channels, particularly TRPA1, TRPV1, TRPV3, and TRPV4, are essential for processing and enhancing these pruritic signals [10,68].

### 4.2. TRP Channels in KCs and FBs Mediate Pruritus

KCs and FBs significantly contribute to pruritus, with specific TRP channels being crucial in this process [67]. TRPV3 is predominantly found in KCs and is activated by its upstream receptor, protease-activated receptor 2 (PAR2). In models of atopic dermatitis in mice, the activation of PAR2 leads to calcium mobilization and the activation of TRPV3, which in turn stimulates the release of TSLP, a strong itch-inducing factor [69]. Elevated levels of PAR2, TRPV3, and TSLP have been observed in human burn tissues, especially in areas experiencing itch. TSLP not only directly triggers pruritus but also promotes mast cell degranulation, which releases tryptase that further activates TRPV3, intensifying the sensation of pruritus [70]. The activation of TRPV4 in KCs results in calcium influx that initiates ERK phosphorylation through the MEK/ERK/MAPK pathway, quickly converting KCs into active cells that promote pruritus [47,59]. Research using mouse models indicates that TRPV4 in KCs plays a role in both acute and chronic pruritus responses [55,71]. Additionally, FBs contribute to pruritus and interact with KCs. Factors like TGF-β1 or histamine can prompt FBs to generate periostin, which induces pruritus through both direct and indirect mechanisms. Directly, periostin interacts with pruriceptive nerve fibers via integrin αVβ3, involving TRPA1 and/or TRPV1 [72,73]. Indirectly, it activates immune cells such as macrophages and eosinophils to release IL-31, which influences KCs to enhance TSLP production [73].

### 4.3. TRP Channels in Immunocytes Mediate Pruritus

Immune cells are a major source of pruritogens within the inflammatory environment of PS [29,39]. Mast cells are particularly significant in the context of pruritus associated with PS, exhibiting higher quantities and degranulation in pruritic scars compared to those without pruritus [74,75]. The expression of TRPA1 is notably elevated in the dermal mast cells of individuals suffering from pruritic skin conditions, indicating its potential role in regulating degranulation and the subsequent release of histamine, tryptase, and IL-31 [76]. Important cytokines released by immune cells contribute to pruritus by acting directly on sensory neurons. IL-4 and IL-13 directly activate TRPA1+ sensory neurons via IL-4Rα, leading to chronic pruritus. Meanwhile, IL-31 initiates acute pruritus by engaging IL-31Rα on TRPV1+ sensory neurons, and TGF-β promotes IL-31 production in dermal conventional type 2 dendritic cells [62]. Additionally, TGF-β can enhance delayed itch by increasing the expression of IL-31Ra, TRPV1, and Nppb, which heightens neuronal sensitivity. This cytokine may also stimulate IL-31 production in dendritic cells, potentially connecting fibrotic and pruritic pathways [77].

### 4.4. TRP Channels in Sensory Nerves Mediate Pruritus

**Figure 3:**
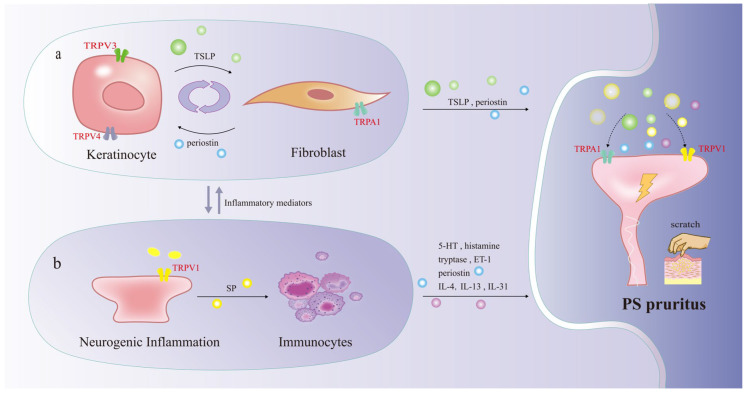
Mechanism network of TRP channels in pathogenic dermal pruritus. Extensive crosstalk is observed among KCs, FBs (a), immunocytes, and sensory nerves (b), which collectively contribute to the pathophysiology of pruritus. TRPA1, TRPV1, TRPV3, and TRPV4 are the main TRP channels involved. TSLP Thymic stromal lymphopoietin, SP substance P, IL-4 interleukin-4, PS pathological scars, TRPA1 transient receptor potential A1.

Neurogenic inflammation refers to the release of inflammatory mediators originating from peripheral afferent neurons like SP and CGRP. The TRPV1 receptor stimulates the release of stored neuropeptides in nerve terminals, which contributes to inflammation and local blood vessel dilation [78,79]. SP is primarily associated with pruritus, directly by binding to neurokinin 1 receptor (NK-1R) and indirectly by amplifying inflammatory responses through interactions with immune cells such as mast cells [80]. This neurogenic mechanism not only sustains pruritus but also intensifies the inflammatory environment, which may further encourage fibrosis [81] (Figure 3).

## 5. TRP Channels as a Therapeutic Target for PS: From Concept to Clinical Translation

### 5.1. TRP Channels as Dual Regulators of Fibrosis and Pruritus

**Figure 4:**
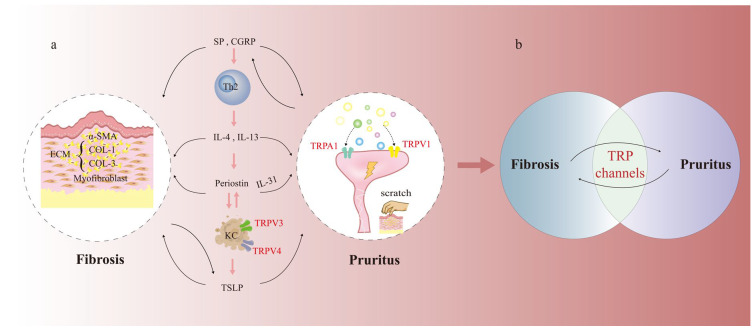
TRP Channels as Dual Regulators of Fibrosis and Pruritus. (**a**) A mechanistic diagram depicting TRP channels as dual regulators of fibrosis and pruritus. (**b**) A simplified schematic illustrating the relationship between TRP channels, fibrosis, and pruritus. SP substance P, GPCRs G protein-coupled receptors, TSLP Thymic stromal lymphopoietin, IL-4 interleukin-4, KC keratinocyte, TRPA1 transient receptor potential A1.

The simultaneous presence of fibrosis and pruritus is a hallmark characteristic of PS, where pruritus serves as a clinical indicator of scar hyperplasia and progression. Although this relationship is widely acknowledged, a comprehensive molecular model that clarifies their concurrent development and provides specific treatment approaches has yet to be established. Recent findings indicate that these apparently separate phenomena may have a shared pathological microenvironment, marked by ongoing inflammatory signals and neuroimmune responses (Figure 4).

Within this integrated microenvironment, specific TRP channels, notably TRPA1, TRPV1, TRPV3, and TRPV4, exhibit a remarkable ability to simultaneously influence both fibrotic and pruritus-related signaling pathways. At the cellular and molecular level, these interactions are highly specific: TRPA1 serves as a nexus for Th2-type inflammation. Cytokines IL-4 and IL-13, primarily produced by Th2 cells and ILC2s (Group 2 Innate Lymphoid Cells), directly sensitize TRPA1 on sensory neurons, promoting pruritus. Simultaneously, these cytokines upregulate TRPA1 in dermal FBs, enhancing their production of periostin, which subsequently promotes TGF-β1 secretion and fibrogenesis [80]. Furthermore, TRPA1 activation in mast cells can modulate the release of pruritogens such as histamine and IL-31. TRPV1 plays a pivotal role in neurogenic signaling; its activation on sensory nerve terminals leads to the release of neuropeptides SP and CGRP. SP acts directly on FBs via NK-1R to stimulate collagen production and on mast cells to perpetuate inflammation and pruritus [54,80,82]. CGRP contributes to a pro-fibrotic microenvironment by skewing immune responses toward a Th17 profile, creating a feedback loop where inflammation activates TRPV1, and TRPV1-mediated neuropeptide release further fuels inflammation and fibrosis [83,84]. TRPV3 and TRPV4, predominantly expressed in KCs, act as sensors for the scar microenvironment. Mechanical stress, heat, or inflammatory mediators activate these channels, triggering the robust production of TSLP. TSLP then acts as a master switch, targeting both TRPA1^+^ sensory neurons to evoke pruritus and FBs via TSLPR to activate Smad2/3 signaling and collagen synthesis, thereby directly linking epidermal sensing to dermal fibrosis [67,69]. This capacity to act as common signaling hubs renders these TRP channels particularly attractive as therapeutic targets for interventions addressing both pathways in PS (Table 2).

Drawing from this information, we put forward an innovative theory: TRP channels, particularly TRPA1, TRPV1, TRPV3, and TRPV4, serve as essential signaling hubs in the PS microenvironment, capable of sensing diverse pathogenic stimuli and concurrently driving both fibrotic and pruritic processes. This integrative role positions them as uniquely promising therapeutic targets for dual-pathway intervention in PS.

### 5.2. Current Management and Unmet Needs in PS

The management of PS in clinical settings continues to encounter obstacles, such as high rates of recurrence and significant variability among patients, underscoring the necessity for a deeper mechanistic insight and more effective treatment options. Current pharmacological treatments mainly focus on targeting fibrotic changes, with relief from pruritus often being a secondary outcome. Corticosteroids administered via intralesional injection, frequently combined with 5-fluorouracil, are the most widely used clinical treatment for PS [86]. Numerous randomized controlled trials and meta-analyses have demonstrated their effectiveness in improving scar vascularity, texture, and height, as well as in alleviating local symptoms like pain and pruritus [87]. Although these therapies can yield favorable outcomes when serious side effects are minimized, their ability to relieve pruritus is frequently short-lived and inconsistent. Additionally, prolonged use of topical corticosteroids can lead to skin-related adverse effects, including pruritus [88]. Other first-line topical agents, such as silicone gel and asiaticoside cream, also aim at fibrotic pathways but show limited success in addressing pruritus [89,90]. Notably, no drugs have been specifically developed and approved to date for pruritus in PS, highlighting a substantial unmet medical need and an opportunity for novel therapeutic strategies.

Emerging evidence suggests that TRP channels may serve as particularly promising therapeutic targets to address this dual therapeutic need. Our previous analysis identified TRPA1, TRPV1, TRPV3, and TRPV4 as compelling candidates based on their involvement in both FB activation and pruriceptive signaling pathways within the scar microenvironment. These channels act as molecular hubs for various harmful stimuli, such as inflammatory cytokines, neurogenic factors, and mechanical pressure. Addressing both fibrosis and pruritus simultaneously marks a significant improvement compared to existing methods that focus on a single pathway.

### 5.3. Current Landscape of TRP-Targeted Drug Development

Significant progress has been made in the development of TRP-targeted treatments for various skin and pain disorders, although the advancement for PS is still in its infancy. A detailed summary of TRP-targeted medications currently undergoing clinical trials can be found in Table 3.

#### 5.3.1. TRPA1-Targeted Drugs

Imiquimod, an immunomodulator not sanctioned for the treatment of PSs, exhibits potential for reducing scarring through mechanisms involving TRPA1. When applied topically, it promotes tissue healing and minimizes the likelihood of scar recurrence after excision by activating TRPA1 in dermal dendritic cells [91], which in turn stimulates the production of IL-23 and activates γδ T cells that secrete IL-17 [108]. U73122, a phospholipase C inhibitor, has recently been identified as a potent and selective TRPA1 agonist capable of inducing TRPA1-dependent CGRP release in murine models [93]. While TRPA1 antagonists show preclinical promise in pain management and respiratory diseases, their clinical application is hindered by issues such as poor aqueous solubility, limited bioavailability, and interspecies variability [109].

#### 5.3.2. TRPV1-Targeted Drugs

TRPV1 was initially identified as the capsaicin receptor. In vitro studies have shown that capsaicin effectively reduces the proliferation of keloid FBs while also depleting SP, which indicates that capsaicin possesses both anti-fibrotic and anti-itch properties [110,111]. The FDA-approved 8% capsaicin patch (Qutenza^®^) is effective for neuropathic pain but has drawbacks, such as temporary burning sensations and potential cancer risks associated with long-term skin accumulation [94]. Next-generation TRPV1 antagonists aim to overcome these limitations. are being developed to address these issues. SAF312, a targeted small-molecule antagonist, is currently in phase II trials for managing postoperative eye pain [95]. The Asivatrep^®^ cream (PAC14028), another selective TRPV1 antagonist, has shown effectiveness in alleviating pruritus in patients with atopic dermatitis [96,97]. Despite their therapeutic potential in pain and pruritus management, TRPV1 antagonist development faces two main challenges: disruptions in systemic temperature regulation and their hydrophobic nature [112].

#### 5.3.3. TRPV2-Targeted Drugs

Tranilast is a notable example of a clinically available agent that interacts with TRP channels. While it was not initially designed to target TRP channels, it has been recognized as an antagonist of the TRPV2 channel and is recommended in clinical protocols for managing keloids in both China and Japan [1,113]. The drug operates by inhibiting TRPV2, which in turn diminishes critical pathways associated with fibrosis, including TGF-β and MAPK signaling. Furthermore, tranilast acts as a prolyl hydroxylase inhibitor, leading to a decrease in collagen production, and its antiallergic effects help to alleviate inflammation [99]. The drug is available in both oral and topical formulations and has demonstrated efficacy in improving symptoms associated with keloids and HSs. It offers particular benefit to patients with extensive scarring, such as those resulting from severe burns [114]. Tranilast established use provides valuable clinical validation for the principle of TRP modulation as a therapeutic strategy in scar management [99].

#### 5.3.4. TRPV3/V4-Targeted Drugs

Recent studies suggest that mutations leading to enhanced function of TRPV3 are linked to skin inflammation and pruritus. KM-001 is a new experimental medication aimed at alleviating pruritus by inhibiting TRPV3. It has demonstrated significant efficacy in reducing pruritus in mice without causing neurobehavioral adverse effects and is currently undergoing trials for conditions such as chronic lichen simplex and palmoplantar keratoderma [100]. Another potential TRPV3 inhibitor for treating inflammatory skin disorders is Trpvicin [115]. Various natural substances derived from plants have been recognized as modulators of TRPV3, including polysaccharide helix [103], forsythoside B [104], coumarin osthole [116], and verbascoside [105]. Additionally, plant-derived compounds, like vitexin [106] and Cimifugin [107], have been noted for their effects on TRPV4 activity. Although the preclinical findings are encouraging, TRPV3 and TRPV4 antagonists are still behind other TRP channels, particularly TRPA1 and TRPV1, with many candidates still in the initial stages of discovery. Translation requires optimization of drug-like properties and rigorous clinical validation.

Despite these advances, it should be emphasized that the potential efficacy of TRP modulation in PS remains hypothetical rather than established. None of these agents have been formally evaluated in rigorous clinical trials specifically designed for PS. The rationale for this strategy is based on two main factors: firstly, the molecular pathways connecting these channels to fibrosis and pruritus, as discussed earlier; and secondly, their proven effectiveness in similar skin disorders like atopic dermatitis and scleroderma models.

### 5.4. Translational Challenges and Future Perspectives

TRP channels present a compelling opportunity for the treatment of PS; however, significant challenges remain in elucidating their mechanisms and in developing effective pharmacological agents. Current research predominantly draws from studies on diseases such as scleroderma, atopic dermatitis, and wound healing, which constrains our understanding of the specific roles that TRP channels play in PS. Notable gaps in our knowledge persist, particularly regarding the unclear expression patterns of TRP channels in various cell types within scar tissue and the distinctions between keloid and HS biology [117]. Additionally, there is a marked deficiency of validated animal models for keloids. Keloids are characterized by unique features, including extension beyond the original wound margins and persistent growth without spontaneous regression. In contrast, scars in traditional animal models, such as rodents and rabbits, typically demonstrate transient hyperplasia confined to the injury site, which resolves spontaneously within weeks. Moreover, so-called ‘keloid-like models,’ such as the transplantation of human keloid tissue into nude mice, depend on immunodeficient animals, which limits the ability to thoroughly investigate the immune system’s role in keloid pathogenesis [118]. Additionally, variations in TRP channel functions across species hinder the applicability of current preclinical findings [89]. Converting these mechanistic insights into viable treatments presents further obstacles. Current TRP modulators frequently exhibit a lack of subtype specificity, and their structural similarities may result in unintended side effects, thereby complicating their clinical application [119]. This issue is primarily due to the high structural homology within the TRP superfamily, where key ligand-binding regions, specifically transmembrane domains 5–6 and the pore region, display a sequence identity of 30–60% across different subtypes [120]. Given the diverse physiological roles of TRP channels, there is a pressing need for innovative delivery methods that can reduce negative side effects, such as loss of thermosensation from TRPV1 inhibition or respiratory issues from TRPA1 blockade [121].

Tackling these interrelated issues demands a collaborative and multidisciplinary strategy that merges fundamental research, drug innovation, and clinical application. Future investigations should utilize advanced methodologies, such as inducible cell-specific knockout models and spatial transcriptomics or single-cell RNA sequencing. These innovative technologies are crucial for pinpointing which channels significantly influence disease advancement at various scar stages (e.g., proliferative versus mature) and types (e.g., keloid versus HS). The absence of standardized animal models, especially for keloids, along with species-specific differences in TRP expression, highlights the need for more physiologically relevant models, including humanized xenograft systems and three-dimensional scar organoids, to enhance preclinical drug assessments [122]. Efforts in drug development should prioritize achieving greater subtype selectivity through structure-based design, leveraging machine learning, computational molecular simulations, and drug repurposing strategies. Investigating advanced localized delivery mechanisms can help sustain therapeutic drug levels within scar tissue while reducing systemic exposure. Any new TRP-targeted treatment must demonstrate clear advantages over existing first-line therapies, whether used alone or in combination. The dual-pathway strategy of TRP modulation—addressing both fibrosis and pruritus—could provide a distinct advantage, potentially allowing for lower corticosteroid dosages and better long-term results. Nonetheless, this theory requires thorough validation through well-structured clinical trials. Future dedicated trials should have dual endpoints for fibrosis and pruritus, and subtype-specific safety monitoring to overcome current limitations of heterogeneity and off-target effects. By adopting this comprehensive approach, which enhances mechanistic insights while tackling challenges in drug development, the field may ultimately translate the theoretical potential of TRP modulation into significant clinical improvements for patients with PS.

## 6. Conclusions

This review establishes a compelling mechanistic framework for TRP channels—specifically TRPA1, TRPV1, TRPV3, and TRPV4—acting as molecular integrators in the PS microenvironment. Their simultaneous expression in KCs, FBs, immune cells, and sensory neurons enables them to coordinate cross-cellular communication and to concurrently regulate both fibrotic remodeling and pruritic signaling, positioning them as promising targets for dual-pathway therapeutic intervention.

The growing field of drug development focused on TRP channels presents a variety of promising options, yet their use in PS is still largely uncharted. Compounds such as the TRPV1 blocker asivatrep and the TRPV3 inhibitor KM-001 have shown significant antipruritic effects in skin disorders, while tranilast, a TRPV2 antagonist, has provided concrete clinical evidence for TRP modulation in treating keloids. Furthermore, several natural substances that target TRPV3 and TRPV4 are progressing through early-stage research. Nonetheless, it is crucial to note that the potential therapeutic benefits of these drugs for PS are still theoretical, as none have undergone thorough evaluation in dedicated clinical trials for PS.

Translating this promise into clinical reality requires addressing several interconnected challenges. The limited subtype selectivity of existing TRP modulators, coupled with significant structural homology among TRP subfamilies, complicates achieving therapeutic specificity. Furthermore, the widespread physiological functions of TRP channels necessitate the development of localized delivery strategies to minimize mechanism-based adverse effects. Demonstrating comparative advantage over established therapies will require clinical trials incorporating co-primary endpoints that simultaneously capture improvements in both scar characteristics and pruritus intensity.

Advancements in the future will rely on collaborative and interdisciplinary approaches. Emphasis must be placed on creating highly specific TRP modulators using structure-oriented design, developing human scar models that accurately reflect physiological conditions for preclinical testing, and introducing novel localized delivery mechanisms. By thoroughly exploring TRP channel biology in genuine PS environments and strategically crafting targeted therapeutic solutions, the field could unlock the benefits of dual-pathway modulation, ultimately offering extensive relief to individuals suffering from PS.

## Figures and Tables

**Table 1:** The main pruritogens and receptors on pruriceptive nerve fibers.

Upstream Receptors		Pruritogens	Source of Pruritogens	Downstream TRP Channels	Refs.
GPCRs	H1R, H4R	Histamine	Mast cell	TRPV1	[47,48]
	B2R	Bradykinin	Mast cell	TRPA1, TRPV1	[49]
	MrgprC11	BAM8-22	KC	TRPA1, TRPV1	[50]
	MrgprC11	Cathepsin S	Macrophagocyte	TRPA1, TRPV1	[51]
	PAR2	Tryptase	Mast cell	TRPA1, TRPV1	[52]
	NK-1R	SP	Nerve terminal	TRPV1	[53,54]
	5-HT1,2,7R	5-HT	Mast cell, KC	TRPA1	[55]
	LTB4R	Leukotriene B4	KC	TRPA1, TRPV1	[56]
	ET-αR	ET-1	Endothelial cell	TRPA1	[57]
Cytokine receptors	IL-7Rα, TSLPR	TSLP	KC	TRPA1	[58,59]
	IL-4Rα, IL-13Rα1	IL-4, IL-13	Th2 cell, ILC2	TRPA1, TRPV1	[60]
	IL-31Rα, OSMR	IL-31	Th2 cell	TRPA1, TRPV1	[61,62,63]
	IL-1RAcP, ST2	IL-33	KC	TRPA1, TRPV1	[38,64]
TLRs	TLR3, TLR4	PIC	TLR Agonist	TRPV1	[65]
	TLR7	Imiquimod	Drug	TRPV1	[66]

Abbreviations: 5-HT, 5-Hydroxytryptamine; BAM8-22, Bovine Adrenal Medulla peptide 8-22; CGRP, Calcitonin Gene-Related Peptide; ET-1, Endothelin-1; GPCR, G Protein-Coupled Receptor; IL, Interleukin; KC, Keratinocyte; LTB4, Leukotriene B4; Mrgpr, Mas-related G protein-coupled receptor; NK-1R, Neurokinin-1 Receptor; PAR2, Protease-Activated Receptor 2; PIC, Poly(I:C); SP, Substance P; TLR, Toll-like Receptor; TRP, Transient Receptor Potential; TSLP, Thymic Stromal Lymphopoietin.

**Table 2:** The Mechanisms TRP channel in dermal fibrosis and pruritus.

Sub-Families	Type	Mechanisms	Refs.
TRPA1	Fibrosis	IL-4/IL-13 activate TRPA1, promoting PS fibrosis via TGF-β/SMAD and IL-4Rα/STAT6 signaling.	[39,80]
		Activation of TRPA1 enhances FBs production of periostin, which then facilitates TGF-β1 secretion via the RhoA/ROCK pathway.	[39]
	Pruritus	IL-4 and IL-13 stimulate TRPA1 neuronal expression and induce pruritus.	[10]
		Activation of TRPA1 stimulates FBs to produce periostin, which subsequently triggers pruritus.	[60,72]
TRPV1	Fibrosis	The SP-NK1R signaling pathway stimulates FBs to secrete COL-1.	[82]
		TRPV1 activation induces FBs to produce periostin, which subsequently regulates TGF-β1 secretion through the RhoA/ROCK pathway.	[39]
		TRPV1 activation triggers the release of CGRP, increases COL-1, TGF-β1, and α-SMA expression, elevates macrophage-related inflammatory factors (IL-1, IL-6, TNF-α, CCL2) through NF-κB and ERK pathways, and enhances IL-17 release from type 17 inflammation.	[78,79]
	Pruritus	SP released by TRPV1 binds to the NK1R receptor, which either mediates neurogenic pruritus or activates Th2 immune cells. This activation leads to the release of IL-4 and IL-13, further promoting pruritus.	[54,80,82]
		TRPV1 activation stimulates FBs to produce periostin, which contributes to the induction of pruritus.	[72,73]
		TRPV1 activation triggers mast cells to release IL-31, which plays a role in inducing pruritus.	[85]
TRPV3	Fibrosis	TRPV3 activation enhances the expression of COL-1, TGF-β1, α-SMA, and fibronectin in FBs via the Smad2/3 signaling pathway.	[32]
	Pruritus	The upregulation of TRPV3 channel increases the expression of TSLP and PAR2 to induce pruritus	[67,69]
TRPV4	Fibrosis	TRPV4 activation may promote FBs differentiation into MFBs by upregulating IL-6.	[9]
	Pruritus	TRPV4 induces KCs to release TSLP to promote pruritus.	[59]

Abbreviations: α-SMA, Alpha-Smooth Muscle Actin; CGRP, Calcitonin Gene-Related Peptide; COL-1, Collagen Type I; EMT, Epithelial-Mesenchymal Transition; FB, Fibroblast; IL, Interleukin; KC, Keratinocyte; NF-κB, Nuclear Factor Kappa-Light-Chain-Enhancer of Activated B Cells; PAR2, Protease-Activated Receptor 2; PS, Pathological Scar; SP, Substance P; TGF-β1, Transforming Growth Factor-Beta 1; Th2, T helper 2 cell; TRP, Transient Receptor Potential; TSLP, Thymic Stromal Lymphopoietin.

**Table 3:** Translational status of TRP-targeted drugs.

TRP Channel	Drug	Mechanisms	Clinical Stage	Indications	Ref
TRPA1	Imiquimod (Aldara^®^, Wilmington, CA, USA)	Immunomodulator activates TLR7/8, suppressing FBs and boosting collagen breakdown via IFN-α. Imiquimod stimulates local IL-23 by activating TRPA1 and activating γδT cells that produce IL-17, promoting tissue regeneration and preventing scar formation.	Approved (FDA)	AK, sBCC, Condylomata Acuminata	[91]
	GRC-17536 (Glenmark, Mumbai, India)	An oral small-molecule TRPA1 antagonist.	Phase II	DPN (pain)	[92]
	U73122 (ENZO, Farmingdale, NY, USA)	A phospholipase C inhibitor. A highly selective TRPA1 agonist induces TRPA1-dependent CGRP release from mouse hand paw skin.	Preclinical	Pruritus	[93]
TRPV1	Capsaicin (Qutenza^®^, Aachen, Germany)	A TRPV1 agonist that reduces pruritus and pain sensitivity by depleting SP.	Approved (FDA)	DPN (pain), PHN	[94]
	Libvatrep/SAF-312	A TRPV1 antagonist.	Phase II	COSP	[95]
	Asivatrep/PAC-14028 (Amorepacific, Seoul, Republic of Korea)	A sulfonamide TRPV1 antagonist exerts anti-inflammatory effects and enhances skin barrier function by inhibiting IL-4/IL-13-mediated JAK/STAT pathways and suppressing neuropeptide SP release.	Phase III	AD (pruritus), Acne Vulgaris	[96,97]
	AG-1529 (AntalGenics, Madrid, Spain)	A modest TRPV1 inhibitor, capsaicin analogs under investigation, dose-dependently attenuates acute histaminergic pruritus in murine model.	Preclinical	Pruritus	[98]
TRPV2	Tranilast (Kissei Pharmaceutical Co, Nagano, Japan)	A TRPV2 antagonist effectively inhibits fibrosis pathways such as TGF-β and MAPK signaling.	Approved (Japan, China, for PS, FDA)	PS (fibrosis), AD, bronchial asthma, allergic rhinitis	[99]
TRPV3	KM-001 (Kamari Pharma, Cambridge, MA, USA)	A TRPV3 inhibitor alleviates mouse skin pruritus by inhibiting TRPV3 activity, without adverse effects on behavior or the nervous system.	Phase I	PPKP1, PC, LSC (pruritus)	[100]
	GRC-15300 (Glenmark, Mumbai, Mumbai, India)	A TRPV3 antagonist.	Phase II	Neuropathic Pain, OA	[101]
	Trpvicin (Sunshine Anjin, Nanjing, China)	A high-affinity TRPV3 antagonist stabilizes TRPV3 in a closed state, minimally affecting other TRPV channels.	Preclinical	AD	[102]
	Polysaccharide Helix (Kwangmyungdang Medicinal Herbs, Ulsan, Republic of Korea)	A plant extract modulates TRPV3 activation and inhibits mast cell degranulation to relieve pruritus and repair the skin barrier.	Preclinical	Pruritus	[103]
	Forsythoside B (Qingya, Wuhan, China)	A plant extract suppresses skin inflammation and pruritus by inhibiting NF-κB signaling and TRPV3 activation.	Preclinical	Pruritus	[104]
	Verbascoside (Qingya, Xi’an, China)	A plant extract inhibits TRPV3 to reduce skin inflammatory factors like TSLP, TNF-α, and IL-6.	Preclinical	Pruritus	[105]
TRPV4	Vitexin (Haoze, Xi’an, China)	A flavonoid compound inhibits TRPV4, reducing inflammatory cell infiltration and alleviating pruritus.	Preclinical	Pruritus	[106]
	Cimifugin (Tauto, Shanghai, China)	The main component of traditional Chinese medicine Fangfeng inhibits TRPV4 and MrgprA3 to exert anti-pruritus effects.	Preclinical	Pruritus	[107]

Abbreviations: AK actinic keratosis, COSP chronic ocular surface pain, DPN diabetic peripheral neuropathy, FB, Fibroblast; IFN-α, Interferon-Alpha; IL, Interleukin; JAK/STAT, Janus Kinase/Signal Transducer and Activator of Transcription; LSC, Lichen Simplex Chronicus; OA, Osteoarthritis; PC, Prurigo Nodularis; PHN, Postherpetic Neuralgia; PPKP1, Punctate Palmoplantar Keratoderma Type 1; PS, Pathological Scars; sBCC, Superficial Basal Cell Carcinoma; SP, Substance P; TGF-β, Transforming Growth Factor-Beta; TLR, Toll-like Receptor; TNF-α,Tumor Necrosis Factor-Alpha; TSLP, Thymic Stromal Lymphopoietin.

## Data Availability

No new data were created or analyzed in this study. Data sharing is not applicable to this article.

## Abbreviations

The following abbreviations are used in this manuscript:

PS	Pathological Scars
TRP	Transient Receptor Potential
KC	Keratinocyte
FB	Fibroblast
MFB	Myofibroblast
ECM	Extracellular Matrix
HS	Hypertrophic Scar
IL	Interleukin
TGF-β1	Transforming Growth Factor-Beta 1
TNF-α	Tumor Necrosis Factor-Alpha
TSLP	Thymic Stromal Lymphopoietin
SP	Substance P
CGRP	Calcitonin Gene-Related Peptide
NF-κB	Nuclear Factor Kappa-Light-Chain-Enhancer of Activated B Cells
MAPK	Mitogen-Activated Protein Kinase
PI3K	Phosphoinositide 3-Kinase
AKT	Protein Kinase B
ERK	Extracellular Signal-Regulated Kinase
JAK	Janus Kinase
STAT	Signal Transducer and Activator of Transcription
Smad	Mothers Against Decapentaplegic Homolog
NFAT	Nuclear Factor of Activated T-cells
YAP	Yes-Associated Protein
TAZ	Transcriptional Co-activator with PDZ-Binding Motif
PAR2	Protease-Activated Receptor 2
EDNRB	Endothelin Receptor Type B
ET-1	Endothelin-1
Ca^2+^	Calcium Ion
α-SMA	Alpha-Smooth Muscle Actin
COL-1	Collagen Type I
COL-3	Collagen Type III
ILC2	Group 2 Innate Lymphoid Cells
EMT	Epithelial-Mesenchymal Transition
RCT	Randomized Controlled Trial
AI	Artificial Intelligence

## References

[1] Ogawa R., Akita S., Akaishi S., Aramaki-Hattori N., Dohi T., Hayashi T., Kishi K., Kono T., Matsumura H., Muneuchi G. (2019). Diagnosis and Treatment of Keloids and Hypertrophic Scars—Japan Scar Workshop Consensus Document 2018. Burns Trauma.

[2] Tracy L.M., Edgar D.W., Schrale R., Cleland H., Gabbe B.J., The BRANZ Adult Long-Term Outcomes Pilot Project Participating Sites and Working Party (2020). Predictors of Itch and Pain in the 12 Months Following Burn Injury: Results from the Burns Registry of Australia and New Zealand (BRANZ) Long-Term Outcomes Project. Burns Trauma.

[3] Kassi K., Kouame K., Kouassi A., Allou A., Kouassi I., Kourouma S., Ecra E., Sangare A. (2020). Quality of Life in Black African Patients with Keloid Scars. Dermatol. Rep..

[4] Bijlard E., Kouwenberg C.A.E., Timman R., Hovius S.E.R., Busschbach J.J.V., Mureau M.A.M. (2017). Burden of Keloid Disease: A Cross-Sectional Health-Related Quality of Life Assessment. Acta Derm. Venereol..

[5] Upton D., Richardson C., Andrews A., Rippon M. (2013). Wound Pruritus: Prevalence, Aetiology and Treatment. J. Wound Care.

[6] Van Der Wal M.B.A., Tuinebreijer W.E., Bloemen M.C.T., Verhaegen P.D.H.M., Middelkoop E., Van Zuijlen P.P.M. (2012). Rasch Analysis of the Patient and Observer Scar Assessment Scale (POSAS) in Burn Scars. Qual. Life Res..

[7] Wang Z.-C., Zhao W.-Y., Cao Y., Liu Y.-Q., Sun Q., Shi P., Cai J.-Q., Shen X.Z., Tan W.-Q. (2020). The Roles of Inflammation in Keloid and Hypertrophic Scars. Front. Immunol..

[8] Ogawa R. (2017). Keloid and Hypertrophic Scars Are the Result of Chronic Inflammation in the Reticular Dermis. Int. J. Mol. Sci..

[9] Okada Y., Sumioka T., Reinach P.S., Miyajima M., Saika S. (2022). Roles of Epithelial and Mesenchymal TRP Channels in Mediating Inflammatory Fibrosis. Front. Immunol..

[10] Shirolkar P., Mishra S.K. (2022). Role of TRP Ion Channels in Pruritus. Neurosci. Lett..

[11] Sun S., Dong X. (2016). Trp Channels and Itch. Semin. Immunopathol..

[12] Mäki-Opas I., Hämäläinen M., Moilanen E., Scotece M. (2023). TRPA1 as a Potential Factor and Drug Target in Scleroderma: Dermal Fibrosis and Alternative Macrophage Activation Are Attenuated in TRPA1-Deficient Mice in Bleomycin-Induced Experimental Model of Scleroderma. Arthritis Res. Ther..

[13] Zheng Y., Huang Q., Zhang Y., Geng L., Wang W., Zhang H., He X., Li Q. (2023). Multimodal Roles of Transient Receptor Potential Channel Activation in Inducing Pathological Tissue Scarification. Front. Immunol..

[14] Kashio M., Tominaga M. (2022). TRP Channels in Thermosensation. Curr. Opin. Neurobiol..

[15] Alaimo A., Rubert J. (2019). The Pivotal Role of TRP Channels in Homeostasis and Diseases throughout the Gastrointestinal Tract. Int. J. Mol. Sci..

[16] Szabó I.L., Herczeg-Lisztes E., Szegedi A., Nemes B., Paus R., Bíró T., Szöllősi A.G. (2019). TRPV4 Is Expressed in Human Hair Follicles and Inhibits Hair Growth In Vitro. J. Investig. Dermatol..

[17] Zhang M., Ma Y., Ye X., Zhang N., Pan L., Wang B. (2023). TRP (Transient Receptor Potential) Ion Channel Family: Structures, Biological Functions and Therapeutic Interventions for Diseases. Sig. Transduct. Target. Ther..

[18] Zhao X., Kwan J.Y.Y., Yip K., Liu P.P., Liu F.-F. (2020). Targeting Metabolic Dysregulation for Fibrosis Therapy. Nat. Rev. Drug Discov..

[19] Henderson N.C., Rieder F., Wynn T.A. (2020). Fibrosis: From Mechanisms to Medicines. Nature.

[20] Hinz B. (2016). The Role of Myofibroblasts in Wound Healing. Curr. Res. Transl. Med..

[21] Shao Y., Guo Z., Yang Y., Liu L., Huang J., Chen Y., Li L., Sun B. (2022). Neutrophil Extracellular Traps Contribute to Myofibroblast Differentiation and Scar Hyperplasia through the Toll-like Receptor 9/Nuclear Factor Kappa-B/Interleukin-6 Pathway. Burns Trauma.

[22] Ishii T., Uchida K., Hata S., Hatta M., Kita T., Miyake Y., Okamura K., Tamaoki S., Ishikawa H., Yamazaki J. (2018). TRPV2 Channel Inhibitors Attenuate Fibroblast Differentiation and Contraction Mediated by Keratinocyte-Derived TGF-Β1 in an in Vitro Wound Healing Model of Rats. J. Dermatol. Sci..

[23] Geiger F., Zeitlmayr S., Staab-Weijnitz C.A., Rajan S., Breit A., Gudermann T., Dietrich A. (2023). An Inhibitory Function of TRPA1 Channels in TGF-Β1–Driven Fibroblast-to-Myofibroblast Differentiation. Am. J. Respir. Cell Mol. Biol..

[24] Zhi Y., Wang H., Huang B., Yan G., Yan L.-Z., Zhang W., Zhang J. (2021). Panax Notoginseng Saponins Suppresses TRPM7 via the PI3K/AKT Pathway to Inhibit Hypertrophic Scar Formation in Vitro. Burns.

[25] Kokubo K., Onodera A., Kiuchi M., Tsuji K., Hirahara K., Nakayama T. (2022). Conventional and Pathogenic Th2 Cells in Inflammation, Tissue Repair, and Fibrosis. Front. Immunol..

[26] Yin G., Zhao C., Pei W. (2022). Crosstalk between Macrophages and Innate Lymphoid Cells (ILCs) in Diseases. Int. Immunopharmacol..

[27] Chen Y., Jin Q., Fu X., Qiao J., Niu F. (2019). Connection between T Regulatory Cell Enrichment and Collagen Deposition in Keloid. Exp. Cell Res..

[28] Yang S., Plotnikov S.V. (2021). Mechanosensitive Regulation of Fibrosis. Cells.

[29] Zhou Y., Hua T., Weng X., Ma D., Li X. (2022). Calcitonin Gene-Related Peptide Alleviates Hypertrophic Scar Formation by Inhibiting the Inflammation. Arch. Dermatol. Res..

[30] Atoyan R., Shander D., Botchkareva N.V. (2009). Non-Neuronal Expression of Transient Receptor Potential Type A1 (TRPA1) in Human Skin. J. Investig. Dermatol..

[31] Russo B., Brembilla N.C., Chizzolini C. (2020). Interplay Between Keratinocytes and Fibroblasts: A Systematic Review Providing a New Angle for Understanding Skin Fibrotic Disorders. Front. Immunol..

[32] Um J., Kang S.Y., Kim H.J., Chung B.Y., Park C.W., Kim H.O. (2020). Transient Receptor Potential Vanilloid-3 (TRPV3) Channel Induces Dermal Fibrosis via the TRPV3/TSLP/Smad2/3 Pathways in Dermal Fibroblasts. J. Dermatol. Sci..

[33] Szöllősi A.G., Vasas N., Angyal Á., Kistamás K., Nánási P.P., Mihály J., Béke G., Herczeg-Lisztes E., Szegedi A., Kawada N. (2018). Activation of TRPV3 Regulates Inflammatory Actions of Human Epidermal Keratinocytes. J. Investig. Dermatol..

[34] Celià-Terrassa T., Kang Y. (2024). How Important Is EMT for Cancer Metastasis?. PLoS Biol..

[35] Hahn J.M., McFarland K.L., Combs K.A., Supp D.M. (2016). Partial Epithelial-Mesenchymal Transition in Keloid Scars: Regulation of Keloid Keratinocyte Gene Expression by Transforming Growth Factor-Β1. Burns Trauma.

[36] Sharma S., Goswami R., Zhang D.X., Rahaman S.O. (2019). TRPV4 Regulates Matrix Stiffness and TGFβ1-induced Epithelial-mesenchymal Transition. J. Cell Mol. Med..

[37] Murata S., Yamanaka M., Taniguchi W., Kajioka D., Suzuki K., Yamada G., Okada Y., Saika S., Yamada H. (2022). Lack of Transient Receptor Potential Ankyrin 1 (TRPA1) Retards Cutaneous Wound Healing in Mice: A Preliminary Study. Biochem. Biophys. Rep..

[38] Meng J., Li Y., Fischer M.J.M., Steinhoff M., Chen W., Wang J. (2021). Th2 Modulation of Transient Receptor Potential Channels: An Unmet Therapeutic Intervention for Atopic Dermatitis. Front. Immunol..

[39] Maeda D., Kubo T., Kiya K., Kawai K., Matsuzaki S., Kobayashi D., Fujiwara T., Katayama T., Hosokawa K. (2019). Periostin Is Induced by IL-4/IL-13 in Dermal Fibroblasts and Promotes RhoA/ROCK Pathway-Mediated TGF-Β1 Secretion in Abnormal Scar Formation. J. Plast. Surg. Hand Surg..

[40] Shin J.U., Kim S.H., Kim H., Noh J.Y., Jin S., Park C.O., Lee W.J., Lee D.W., Lee J.H., Lee K.H. (2016). TSLP Is a Potential Initiator of Collagen Synthesis and an Activator of CXCR4/SDF-1 Axis in Keloid Pathogenesis. J. Investig. Dermatol..

[41] Ishise H., Larson B., Hirata Y., Fujiwara T., Nishimoto S., Kubo T., Matsuda K., Kanazawa S., Sotsuka Y., Fujita K. (2015). Hypertrophic Scar Contracture Is Mediated by the TRPC3 Mechanical Force Transducer via NFkB Activation. Sci. Rep..

[42] Kawai K., Ishise H., Kubo T., Larson B., Fujiwara T., Nishimoto S., Kakibuchi M. (2023). Stretching Promotes Wound Contraction Through Enhanced Expression of Endothelin Receptor B and TRPC3 in Fibroblasts. Plast. Reconstr. Surg. Glob. Open.

[43] Davis J., Burr A.R., Davis G.F., Birnbaumer L., Molkentin J.D. (2012). A TRPC6-Dependent Pathway for Myofibroblast Transdifferentiation and Wound Healing In Vivo. Dev. Cell.

[44] He J., Fang B., Shan S., Xie Y., Wang C., Zhang Y., Zhang X., Li Q. (2021). Mechanical Stretch Promotes Hypertrophic Scar Formation through Mechanically Activated Cation Channel Piezo1. Cell Death Dis..

[45] Yosipovitch G., Rosen J.D., Hashimoto T. (2018). Itch: From Mechanism to (Novel) Therapeutic Approaches. J. Allergy Clin. Immunol..

[46] Feng J., Zhao Y., Xie Z., Zang K., Sviben S., Hu X., Fitzpatrick J.A.J., Wen L., Liu Y., Wang T. (2022). Miswiring of Merkel Cell and Pruriceptive C Fiber Drives the Itch-Scratch Cycle. Sci. Transl. Med..

[47] Chen Y., Fang Q., Wang Z., Zhang J.Y., MacLeod A.S., Hall R.P., Liedtke W.B. (2016). Transient Receptor Potential Vanilloid 4 Ion Channel Functions as a Pruriceptor in Epidermal Keratinocytes to Evoke Histaminergic Itch. J. Biol. Chem..

[48] Kim B.M., Lee S.H., Shim W.S., Oh U. (2004). Histamine-Induced Ca^2+^ Influx via the PLA2/Lipoxygenase/TRPV1 Pathway in Rat Sensory Neurons. Neurosci. Lett..

[49] Costa R., Manjavachi M.N., Motta E.M., Marotta D.M., Juliano L., Torres H.A., Pesquero J.B., Calixto J.B. (2010). The Role of Kinin B1 and B2 Receptors in the Scratching Behaviour Induced by Proteinase-Activated Receptor-2 Agonists in Mice. Br. J. Pharmacol..

[50] Aliotta G.E., Lo Vecchio S., Elberling J., Arendt-Nielsen L. (2022). Evaluation of Itch and Pain Induced by Bovine Adrenal Medulla (BAM)8-22, a New Human Model of Non-Histaminergic Itch. Exp. Dermatol..

[51] Reddy V.B., Shimada S.G., Sikand P., Lamotte R.H., Lerner E.A. (2010). Cathepsin S Elicits Itch and Signals via Protease-Activated Receptors. J. Investig. Dermatol..

[52] Leal E.C., Carvalho E., Tellechea A., Kafanas A., Tecilazich F., Kearney C., Kuchibhotla S., Auster M.E., Kokkotou E., Mooney D.J. (2015). Substance P Promotes Wound Healing in Diabetes by Modulating Inflammation and Macrophage Phenotype. Am. J. Pathol..

[53] Liu J.-Y., Hu J.-H., Zhu Q.-G., Li F.-Q., Sun H.-J. (2006). Substance P Receptor Expression in Human Skin Keratinocytes and Fibroblasts. Br. J. Dermatol..

[54] Azimi E., Reddy V.B., Pereira P.J.S., Talbot S., Woolf C.J., Lerner E.A. (2017). Substance P Activates Mas-Related G Protein-Coupled Receptors to Induce Itch. J. Allergy Clin. Immunol..

[55] Akiyama T., Ivanov M., Nagamine M., Davoodi A., Carstens M.I., Ikoma A., Cevikbas F., Kempkes C., Buddenkotte J., Steinhoff M. (2016). Involvement of TRPV4 in Serotonin-Evoked Scratching. J. Investig. Dermatol..

[56] Andoh T., Yageta Y., Takeshima H., Kuraishi Y. (2004). Intradermal Nociceptin Elicits Itch-Associated Responses Through Leukotriene B4 in Mice. J. Investig. Dermatol..

[57] Kido-Nakahara M., Buddenkotte J., Kempkes C., Ikoma A., Cevikbas F., Akiyama T., Nunes F., Seeliger S., Hasdemir B., Mess C. (2014). Neural Peptidase Endothelin-Converting Enzyme 1 Regulates Endothelin 1–Induced Pruritus. J. Clin. Investig..

[58] Wilson S.R., Thé L., Batia L.M., Beattie K., Katibah G.E., McClain S.P., Pellegrino M., Estandian D.M., Bautista D.M. (2013). The Epithelial Cell-Derived Atopic Dermatitis Cytokine TSLP Activates Neurons to Induce Itch. Cell.

[59] Lee W.-J., Shim W.-S. (2021). Cutaneous Neuroimmune Interactions of TSLP and TRPV4 Play Pivotal Roles in Dry Skin-Induced Pruritus. Front. Immunol..

[60] Garcovich S., Maurelli M., Gisondi P., Peris K., Yosipovitch G., Girolomoni G. (2021). Pruritus as a Distinctive Feature of Type 2 Inflammation. Vaccines.

[61] Niyonsaba F., Ushio H., Hara M., Yokoi H., Tominaga M., Takamori K., Kajiwara N., Saito H., Nagaoka I., Ogawa H. (2010). Antimicrobial Peptides Human Beta-Defensins and Cathelicidin LL-37 Induce the Secretion of a Pruritogenic Cytokine IL-31 by Human Mast Cells. J. Immunol..

[62] Cevikbas F., Wang X., Akiyama T., Kempkes C., Savinko T., Antal A., Kukova G., Buhl T., Ikoma A., Buddenkotte J. (2014). A Sensory Neuron-Expressed IL-31 Receptor Mediates T Helper Cell-Dependent Itch: Involvement of TRPV1 and TRPA1. J. Allergy Clin. Immunol..

[63] Arai I., Tsuji M., Takeda H., Akiyama N., Saito S. (2013). A Single Dose of Interleukin-31 (IL-31) Causes Continuous Itch-Associated Scratching Behaviour in Mice. Exp. Dermatol..

[64] Trier A.M., Ver Heul A.M., Fredman A., Le V., Wang Z., Auyeung K., Meixiong J., Lovato P., Holtzman M.J., Wang F. (2024). IL-33 Potentiates Histaminergic Itch. J. Allergy Clin. Immunol..

[65] Szöllősi A.G., McDonald I., Szabó I.L., Meng J., van den Bogaard E., Steinhoff M. (2019). TLR3 in Chronic Human Itch: A Keratinocyte-Associated Mechanism of Peripheral Itch Sensitization. J. Investig. Dermatol..

[66] Liu T., Xu Z.-Z., Park C.-K., Berta T., Ji R.-R. (2010). Toll-like Receptor 7 Mediates Pruritus. Nat. Neurosci..

[67] Lin S., Liu X., Jiang J., Ge W., Zhang Y., Li F., Tao Q., Liu S., Li M., Chen H. (2024). The Involvement of Keratinocytes in Pruritus of Chronic Inflammatory Dermatosis. Exp. Dermatol..

[68] Grigore A., Coman O.A., Păunescu H., Costescu M., Fulga I. (2024). Latest Insights into the In Vivo Studies in Murine Regarding the Role of TRP Channels in Wound Healing—A Review. Int. J. Mol. Sci..

[69] Zhao J., Munanairi A., Liu X.-Y., Zhang J., Hu L., Hu M., Bu D., Liu L., Xie Z., Kim B.S. (2020). PAR2 Mediates Itch via TRPV3 Signaling in Keratinocytes. J. Investig. Dermatol..

[70] Park C., Kim H., Choi Y., Chung B., Woo S.-Y., Song D.-K., Kim H. (2017). TRPV3 Channel in Keratinocytes in Scars with Post-Burn Pruritus. Int. J. Mol. Sci..

[71] Luo J., Feng J., Yu G., Yang P., Mack M.R., Du J., Yu W., Qian A., Zhang Y., Liu S. (2018). TRPV4-Expressing Macrophages and Keratinocytes Contribute Differentially to Allergic and Non-Allergic Chronic Itch. J. Allergy Clin. Immunol..

[72] Mishra S.K., Wheeler J.J., Pitake S., Ding H., Jiang C., Fukuyama T., Paps J.S., Ralph P., Coyne J., Parkington M. (2020). Periostin Activation of Integrin Receptors on Sensory Neurons Induces Allergic Itch. Cell Rep..

[73] Hashimoto T., Mishra S.K., Olivry T., Yosipovitch G. (2021). Periostin, an Emerging Player in Itch Sensation. J. Investig. Dermatol..

[74] Siiskonen H., Harvima I. (2019). Mast Cells and Sensory Nerves Contribute to Neurogenic Inflammation and Pruritus in Chronic Skin Inflammation. Front. Cell. Neurosci..

[75] Shao Y., Wang D., Zhu Y., Xiao Z., Jin T., Peng L., Shen Y., Tang H. (2023). Molecular Mechanisms of Pruritus in Prurigo Nodularis. Front. Immunol..

[76] Yin S., Luo J., Qian A., Du J., Yang Q., Zhou S., Yu W., Du G., Clark R.B., Walters E.T. (2013). Retinoids Activate the Irritant Receptor TRPV1 and Produce Sensory Hypersensitivity. J. Clin. Investig..

[77] Xu J., Zanvit P., Hu L., Tseng P.-Y., Liu N., Wang F., Liu O., Zhang D., Jin W., Guo N. (2020). The Cytokine TGF-β Induces Interleukin-31 Expression from Dermal Dendritic Cells to Activate Sensory Neurons and Stimulate Wound Itching. Immunity.

[78] Spekker E., Körtési T., Vécsei L. (2022). TRP Channels: Recent Development in Translational Research and Potential Therapeutic Targets in Migraine. Int. J. Mol. Sci..

[79] Pinho-Ribeiro F.A., Baddal B., Haarsma R., O’Seaghdha M., Yang N.J., Blake K.J., Portley M., Verri W.A., Dale J.B., Wessels M.R. (2018). Blocking Neuronal Signaling to Immune Cells Treats Streptococcal Invasive Infection. Cell.

[80] Nguyen J.K., Austin E., Huang A., Mamalis A., Jagdeo J. (2020). The IL-4/IL-13 Axis in Skin Fibrosis and Scarring: Mechanistic Concepts and Therapeutic Targets. Arch. Dermatol. Res..

[81] Tang L., Gao J., Cao X., Chen L., Wang H., Ding H. (2022). TRPV1 Mediates Itch-Associated Scratching and Skin Barrier Dysfunction in DNFB-Induced Atopic Dermatitis Mice. Exp. Dermatol..

[82] Dong J., Feng F., Xu G., Zhang H., Hong L., Yang J. (2015). Elevated SP/NK-1R in Esophageal Carcinoma Promotes Esophageal Carcinoma Cell Proliferation and Migration. Gene.

[83] Ständer S., Luger T., Kim B., Lerner E., Metz M., Adiri R., Canosa J.M., Cha A., Yosipovitch G. (2024). Cutaneous Components Leading to Pruritus, Pain, and Neurosensitivity in Atopic Dermatitis: A Narrative Review. Dermatol. Ther..

[84] Yook H.J., Lee J.H. (2024). Prurigo Nodularis: Pathogenesis and the Horizon of Potential Therapeutics. Int. J. Mol. Sci..

[85] Lee M.Y., Shin E., Kim H., Kwak I.S., Choi Y. (2018). Interleukin-31, Interleukin-31RA, and OSMR Expression Levels in Post-Burn Hypertrophic Scars. J. Pathol. Transl. Med..

[86] Ehsani A., Lotfi F., Firooz A., Ehsani A., Razavi Z., Ansari M.S., Nourmohammadpour P., Sima F., Koohian Mohammadabadi M., Rahimnia A. (2025). Unlocking Better Keloid Treatment: Corticosteroid, 5-Fluorouracil, and Hyaluronidase vs. Corticosteroid Alone—A Randomized Comparative Study. Health Sci. Rep..

[87] Sheng M., Chen Y., Li H., Zhang Y., Zhang Z. (2023). The Application of Corticosteroids for Pathological Scar Prevention and Treatment: Current Review and Update. Burns Trauma.

[88] Coondoo A., Phiske M., Verma S., Lahiri K. (2014). Side-Effects of Topical Steroids: A Long Overdue Revisit. Indian Dermatol. Online J..

[89] Lindsay C., Timperley C. (2020). TRPA1 and Issues Relating to Animal Model Selection for Extrapolating Toxicity Data to Humans. Hum. Exp. Toxicol..

[90] Li L., Liu L., Wang T. (2025). Effects of Asiaticosides on Scar Recovery and Psychological Well-Being in Patients with Scald Injuries. Pharmacogn. Mag..

[91] Kemény Á., Kodji X., Horváth S., Komlódi R., Szőke É., Sándor Z., Perkecz A., Gyömörei C., Sétáló G., Kelemen B. (2018). TRPA1 Acts in a Protective Manner in Imiquimod-Induced Psoriasiform Dermatitis in Mice. J. Investig. Dermatol..

[92] Mukhopadhyay I., Kulkarni A., Aranake S., Karnik P., Shetty M., Thorat S., Ghosh I., Wale D., Bhosale V., Khairatkar-Joshi N. (2014). Transient Receptor Potential Ankyrin 1 Receptor Activation In Vitro and In Vivo by Pro-Tussive Agents: GRC 17536 as a Promising Anti-Tussive Therapeutic. PLoS ONE.

[93] Neacsu C., Sauer S.K., Reeh P.W., Babes A. (2020). The Phospholipase C Inhibitor U73122 Is a Potent Agonist of the Polymodal Transient Receptor Potential Ankyrin Type 1 (TRPA1) Receptor Channel. Naunyn-Schmiedeberg’s Arch. Pharmacol..

[94] Bonezzi C., Costantini A., Cruccu G., Fornasari D.M.M., Guardamagna V., Palmieri V., Polati E., Zini P., Dickenson A.H. (2020). Capsaicin 8% Dermal Patch in Clinical Practice: An Expert Opinion. Expert Opin. Pharmacother..

[95] Fan J., Ke H., Lei J., Wang J., Tominaga M., Lei X. (2024). Structural Basis of TRPV1 Inhibition by SAF312 and Cholesterol. Nat. Commun..

[96] Lee J.-H., Choi C.S., Bae I.-H., Choi J.K., Park Y.-H., Park M. (2018). A Novel, Topical, Nonsteroidal, TRPV1 Antagonist, PAC-14028 Cream Improves Skin Barrier Function and Exerts Anti-Inflammatory Action through Modulating Epidermal Differentiation Markers and Suppressing Th2 Cytokines in Atopic Dermatitis. J. Dermatol. Sci..

[97] Park C.W., Kim B.J., Lee Y.W., Won C., Park C.O., Chung B.Y., Lee D.H., Jung K., Nam H.-J., Choi G. (2022). Asivatrep, a TRPV1 Antagonist, for the Topical Treatment of Atopic Dermatitis: Phase 3, Randomized, Vehicle-Controlled Study (CAPTAIN-AD). J. Allergy Clin. Immunol..

[98] Nikolaeva-Koleva M., Butron L., González-Rodríguez S., Devesa I., Valente P., Serafini M., Genazzani A.A., Pirali T., Ballester G.F., Fernández-Carvajal A. (2021). A Capsaicinoid-Based Soft Drug, AG1529, for Attenuating TRPV1-Mediated Histaminergic and Inflammatory Sensory Neuron Excitability. Sci. Rep..

[99] Massoud G., Parish M., Hazimeh D., Moukarzel P., Singh B., Cayton Vaught K.C., Segars J., Islam M.S. (2024). Unlocking the Potential of Tranilast: Targeting Fibrotic Signaling Pathways for Therapeutic Benefit. Int. Immunopharmacol..

[100] Barak S.L., Michel D., Brener E., Braiman L., Yosipovitch G. (2023). 629 Evaluating the Safety and Efficacy of Topical KM-001, a TRPV3 Inhibitor, for Treatment of Pruritus in Patients with Lichen Simplex Chronicus (LSC): A First in Human, Double-Blind, Randomized Vehicle-Controlled Study. J. Investig. Dermatol..

[101] Asiri Y.I., Zaheen Hassan M. (2023). An Overview of Ion Channels Therapeutics in the Treatment of Pain. Arab. J. Chem..

[102] Broad L.M., Mogg A.J., Eberle E., Tolley M., Li D.L., Knopp K.L. (2016). TRPV3 in Drug Development. Pharmaceuticals.

[103] Nam J.H., Jung H.W., Chin Y.-W., Yang W.-M., Bae H.S., Kim W.K. (2017). Spirodela Polyrhiza Extract Modulates the Activation of Atopic Dermatitis-Related Ion Channels, Orai1 and TRPV3, and Inhibits Mast Cell Degranulation. Pharm. Biol..

[104] Zhang H., Sun X., Qi H., Ma Q., Zhou Q., Wang W., Wang K. (2019). Pharmacological Inhibition of the Temperature-Sensitive and Ca^2+^-Permeable Transient Receptor Potential Vanilloid TRPV3 Channel by Natural Forsythoside B Attenuates Pruritus and Cytotoxicity of Keratinocytes. J. Pharmacol. Exp. Ther..

[105] Sun X., Qi H., Wu H., Qu Y., Wang K. (2020). Anti-Pruritic and Anti-Inflammatory Effects of Natural Verbascoside through Selective Inhibition of Temperature-Sensitive Ca^2+^-Permeable TRPV3 Channel. J. Dermatol. Sci..

[106] Qin Z., Xiang L., Zheng S., Zhao Y., Qin Y., Zhang L., Zhou L. (2023). Vitexin Inhibits Pain and Itch Behavior via Modulating TRPV4 Activity in Mice. Biomed. Pharmacother..

[107] Yan J., Ye F., Ju Y., Wang D., Chen J., Zhang X., Yin Z., Wang C., Yang Y., Zhu C. (2021). Cimifugin Relieves Pruritus in Psoriasis by Inhibiting TRPV4. Cell Calcium.

[108] Wei J.J., Kim H.S., Spencer C.A., Brennan-Crispi D., Zheng Y., Johnson N.M., Rosenbach M., Miller C., Leung D.H., Cotsarelis G. (2020). Activation of TRPA1 Nociceptor Promotes Systemic Adult Mammalian Skin Regeneration. Sci. Immunol..

[109] Anand S., Rajagopal S. (2023). A Comprehensive Review on the Regulatory Action of TRP Channels: A Potential Therapeutic Target for Nociceptive Pain. J. Exp. Neurosci..

[110] Reyes-Escogido M.D.L., Gonzalez-Mondragon E.G., Vazquez-Tzompantzi E. (2011). Chemical and Pharmacological Aspects of Capsaicin. Molecules.

[111] Abdel-Salam O.M.E., Mózsik G. (2023). Capsaicin, The Vanilloid Receptor TRPV1 Agonist in Neuroprotection: Mechanisms Involved and Significance. Neurochem. Res..

[112] Wang X., Bao C., Li Z., Yue L., Hu L. (2022). Side Effects of Opioids Are Ameliorated by Regulating TRPV1 Receptors. Int. J. Environ. Res. Public Health.

[113] Expert Group of the Standing Committee of the Scar Medicine Branch of the Chinese Association of Plastic and Aesthetic Surgery (2018). Recommended Guidelines for Clinical Treatment of Keloids in China. Chin. J. Aesthetic Plast. Surg..

[114] Darakhshan S., Pour A.B. (2015). Tranilast: A Review of Its Therapeutic Applications. Pharmacol. Res..

[115] Fan J., Hu L., Yue Z., Liao D., Guo F., Ke H., Jiang D., Yang Y., Lei X. (2023). Structural Basis of TRPV3 Inhibition by an Antagonist. Nat. Chem. Biol..

[116] Sun X.-Y., Sun L.-L., Qi H., Gao Q., Wang G.-X., Wei N.-N., Wang K. (2018). Antipruritic Effect of Natural Coumarin Osthole through Selective Inhibition of Thermosensitive TRPV3 Channel in the Skin. Mol. Pharmacol..

[117] Caterina M., Pang Z. (2016). TRP Channels in Skin Biology and Pathophysiology. Pharmaceuticals.

[118] Supp D.M. (2019). Animal Models for Studies of Keloid Scarring. Adv. Wound Care.

[119] Chen X., Sooch G., Demaree I.S., White F.A., Obukhov A.G. (2020). Transient Receptor Potential Canonical (TRPC) Channels: Then and Now. Cells.

[120] Yelshanskaya M.V., Sobolevsky A.I. (2022). Ligand-Binding Sites in Vanilloid-Subtype TRP Channels. Front. Pharmacol..

[121] Demartini L., Bonezzi C. (2025). Neuropathic Pain: Proposal of a Mechanism-Based Treatment. Explor. Neurosci..

[122] Du G., Tian Y., Yao Z., Vu S., Zheng J., Chai L., Wang K., Yang S. (2020). A Specialized Pore Turret in the Mammalian Cation Channel TRPV1 Is Responsible for Distinct and Species-Specific Heat Activation Thresholds. J. Biol. Chem..

